# Colorectal Cancer in Iran: Molecular Epidemiology and Screening Strategies

**DOI:** 10.1155/2015/643020

**Published:** 2015-01-15

**Authors:** Roya Dolatkhah, Mohammad Hossein Somi, Mortaza Jabbarpour Bonyadi, Iraj Asvadi Kermani, Faris Farassati, Saeed Dastgiri

**Affiliations:** ^1^Liver and Gastrointestinal Diseases Research Center, Tabriz University of Medical Sciences, Tabriz, Iran; ^2^Hematology and Oncology Research Center, Tabriz University of Medical Sciences, Tabriz, Iran; ^3^Center of Excellent for Biodiversity, Faculty of Natural Sciences, University of Tabriz, Tabriz, Iran; ^4^Department of Medicine, The University of Kansas Medical School, Molecular Medicine Laboratory, KUMC, Kansas City, KS, USA

## Abstract

*Purpose*. The increasing incidence of colorectal cancer (CRC) in the past three decades in Iran has made it a major public health burden. This study aimed to report its epidemiologic features, molecular genetic aspects, survival, heredity, and screening pattern in Iran. *Methods*. A comprehensive literature review was conducted to identify the relevant published articles. We used medical subject headings, including colorectal cancer, molecular genetics, *KRAS* and *BRAF* mutations, screening, survival, epidemiologic study, and Iran. *Results*. Age standardized incidence rate of Iranian CRCs was 11.6 and 10.5 for men and women, respectively. Overall five-year survival rate was 41%, and the proportion of CRC among the younger age group was higher than that of western countries. Depending on ethnicity, geographical region, dietary, and genetic predisposition, mutation genes were considerably diverse and distinct among CRCs across Iran. The high occurrence of CRC in records of relatives of CRC patients showed that family history of CRC was more common among young CRCs. *Conclusion*. Appropriate screening strategies for CRC which is amenable to early detection through screening, especially in relatives of CRCs, should be considered as the first step in CRC screening programs.

## 1. Introduction and Background

Cancer is a major health problem worldwide and imposes a great economic and psychological burden in addition to loss of life and fertility. Colorectal cancer (CRC) is one of the most common types of cancer affecting 1.23 million individuals per year (9.7% of overall cancers) and is the fourth most common cause of death from cancer worldwide (608,000 cases, 8% of overall cancer deaths). The incidence of CRC has been estimated to be 30–50 cases (per 100,000) in northern America and Europe and 3–7 cases (per 100,000) in most Middle East countries. Although epidemiology data show a marked variability around the world, and almost 60% of cases occur in developed countries, its overall incidence rate shows a slow but steady decrease (about 2% per year) in developed countries. Conversely, in developing countries and most of Asian ones, the annual incidence is unfortunately expected to increase over the next two decades. However, the proportion of CRC cases occurring among younger age group (≤40 years) is 2–8 percent in western countries where it is about 15–35 percent in the Middle East region [1–3].

A large number of epidemiologic studies have been performed to investigate specific hypotheses about risk factors of colorectal cancer, particularly for diet, as western diet (high meat and energy and less fruit, vegetable, and fiber intake), which may have a role in G × E (gene and environmental) interaction [4]. Some approaches specifically reveal a correlation between folate and the* MTHFR* (methylenetetrahydrofolate reductase) gene in red meat with a series of metabolic genes. According to WHO Classification of Tumors of the Digestive System, the following factors may play an etiologic role in the development of CRC, including sedentary lifestyle and obesity, high body and abdominal fatness, hormone replacement therapy, tobacco smoking, alcohol, and nonsteroidal anti-inflammatory drugs [4–6]. However, polyps and high risk bowel syndromes such as inflammatory bowel diseases, especially ulcerative colitis, must be noted as a risk factor for CRC in general population [7].

It has been proved that about 25% of CRC patients have some degrees of familial background, and a strong family history as a first or second degree relation is seen in about 15% of other patients with colorectal cancer [8]. Sporadic or nonhereditary colorectal cancer occurs in people who have no (or very little) family history of CRC and in patients who have no identifiable genetic risk. The majority of CRCs (70–85%) are sporadic and they are believed to be influenced by diet, lifestyle, environmental factors, and acquired somatic mutations [2, 6]. Its characteristics are the stepwise progression from normal epithelium to carcinoma, associated with sequential molecular abnormalities in each step [8]. The origin of the disease may be attributed to the presence of a large number of genetic variants that may act according to a polygenic model. However, nongenetic factors also clearly play a role in sporadic CRCs [3].

It is now recognized that there are two distinct forms of familial colorectal cancer syndrome, whose inheritance is diagnosed as autosomal dominant trait. In familial adenomatous polyposis (FAP) and its subspecies, Gardner syndrome, at risk individuals develop thousands of polyps over the course of their lives. The reported incidence of familial polyposis varies from 1 in 7,000 to 1 in 22,000 individuals. Virtually all patients with FAP will develop CRC at a medium age of about 40, and, because of the diffuse nature of the polyposis, the best way for prevention of CRC is surgical therapy [3, 9–11].

In contrast, hereditary nonpolyposis colorectal cancer (HNPCC) could be defined in terms of the pattern of familial aggregation. Its frequency is about one in 10,000, and it includes almost 2–4% of colorectal cancers. HNPCC is a group of five familial cancer syndromes (HNPCC 1 to HNPCC 5), which occur due to mutation in 5 independent genes involved in DNA mismatch repair. The same genes might be involved in the etiology of both hereditary and sporadic cancers, with the difference that, in hereditary cancers, one copy of a mutant allele is inherited in the germline and the other mutation occurs somatically, while in sporadic cancers, both mutations are acquired somatically [3, 9–12].

Over the past two decades, molecular studies have led to a tremendous increase in our knowledge of genetic changes that affect malignancy in CRC. This has enabled us to better characterize tumors individually and classify them according to certain molecular or genetic features [13]. However, mutation detection methods with high sensitivity will increase the possibility of choosing the correct individual therapy and reduce the risk of metastasis in low stage diseases. A better understanding of the causality of CRC can be established by combining epidemiology and research on molecular mechanisms.

The pathogenesis of colorectal cancer is complex and diverse, with different molecular pathways, and in fact leading to different phenotypes. CRC develops through a series of events that lead to the transformation of normal epithelium to adenoma and then to carcinoma. Three major genetic pathways are implicated in colorectal cancer.

### 1.1. *CIN* (Chromosomal Instability) Pathway

It is the most common type of CRC pathways, which accounts for 85% of all CRCs and 65–70% of sporadic colorectal cancer. This pathway includes the following:

- activation of protooncogenes by mutation, including* KRAS *(Kirsten rat sarcoma viral oncogene homolog 0),* c-Src *(tyrosine-protein kinase* Src*), and* c-Myc *(avian myelocytomatosis viral oncogene homolog),
- inactivation of at least three tumor suppressor genes, as* APC *(adenomatous polyposis coli), tumor suppressor* TP53* gene, and subsequent* LOH *(loss of heterozygosity) of chromosome 18q.

### 1.2. *CIMP* (CPG Island Methylator Phenotype) and the “Serrated” Pathway

The term “serrated” is attributed to morphological serrated appearance or precursor region. This pathway is driven by hypermethylation of genes and is characterized by the presence of protooncogene* BRAF *(protein kinase* B-Raf*), which causes increased* MAPKs/ERKs* (mitogen activated protein kinases/extracellular signal-regulated kinases) signaling, leading to increased cell proliferation, cell division, and secretion [2].

**Figure 1:**
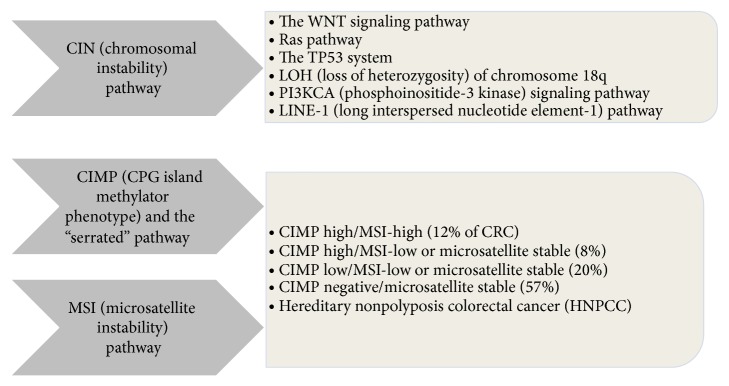
Classifying CRC based on the presence of MSI and CIMP [10].

*CIMP* is found in about 20–30% of colorectal cancers, based on the number of methylator markers, and can be divided into five subgroups (Figure 1).

### 1.3. *MSI *(Microsatellite Instability) Pathway

This pathway is involved in the genesis of about 10–15% of sporadic and >90% of HNPCC syndrome. Instability of microsatellites is a reflection of genetic hypermutability or somatic inactivation that results from inactivation of DNA mismatch repair (*MMR*) genes, including* hMLH1* (human* MutL* homolog 1),* hMSH2* (human* MutS* homologue 2),* hMSH6*, and* hPMS2* (human postmeiotic segregation 2) [2, 14, 15].

Clinically, CRCs are classified as* MSI-H* (high level MSI, cancers with instability at around 30–40% of markers),* MSI-L* (low level* MSI*, cancers with instability less than 30–40% of markers), and* MSS* (microsatellite stable, cancers with no apparent instability) based on the extent of* MSI*. This inactivation is caused by epigenetic silencing via promoter hypermethylation.* BRAF *mutation is frequently seen in sporadic* MSI-H* CRC, but not in HNPCC. Indeed, in sporadic settings,* MSI-H* CRCs are not mostly due to epigenetic silencing of the* hMLH1* gene promoter [14, 15].* MSI* pathway can be considered an early event in the adenoma carcinoma sequence and in ulcerative colitis associated neoplasia [7].

Targeted cancer therapy is becoming a powerful strategy for the treatment of patients selected on the basis of their molecular characteristics. This is particularly true for patients with metastatic CRC. A mutant* KRAS* gene has been associated with resistance to immunotherapy using monoclonal anti-*EGFR* (epidermal growth factor receptor) antibodies (i.e., Cetuximab and Panitumumab). The clinical diagnosis of metastatic CRC currently involves screening of tumor tissue for* KRAS* mutations so that patients with wild-type* KRAS*-containing tumors are only given anti-*EGFR* treatment [16]. In fact, patients with wild-type* KRAS* often have better response with Cetuximab while those with* KRAS* mutation or with tumors of unknown* KRAS* gene orientations are often responsive to Bevacizumab [17]. Targeted anti-*EGFR* treatment has led to a considerable improvement in disease control and survival rates. Selection of patients would therefore show positive therapeutic response. For this reason,* KRAS* and* BRAF* tests are now used for patient selection [18]. Information pertaining to* KRAS* mutation status in patients appears to be effective in choosing target factors for therapy. A survey conducted in 14 European, Latin American, and Asian countries revealed increasing use of* KRAS* test from 3 percent in 2008 to 69 percent in 2010 [17].

Screening strategies include the diagnosis of precancerous polyps and cancer in asymptomatic individuals which may help the prevention of malignancy [19, 20]. Previous studies have shown that use of screening strategies can reduce about 60 percent of the deaths resulting from CRC [20, 21]. Moreover, the administration of CRC screening programs in the United States has led to considerable reduction in cancer-associated death rates. Current regulations suggest that screening for CRC should be initiated in the general population for 50 years old and more, and for the high risk group from a younger age [20]. The regulations for prevention of diseases in the United States require that every man or woman aged 50 years or above should be screened for CRC using one of the screening tests including occult blood test annually, sigmoidoscopy every 5 years, barium enema every 5 years, or colonoscopy every 10 years. Screening is performed using the colonoscopy as a gold standard to determine the prognosis of adenoma of the colon and CRC. Previous studies have shown that the use of colonoscopy for screening for CRC results in reduced danger of cancer-related mortality due to the removal of precancerous polyps. Given that family members of CRC patients are at increased risk for the disease, screening through colonoscopy is usually recommended for the closest relatives (father, mother, sister, brother, and children) of patients with CRC or adenomatous polyps. The new regulations propose that screening methods should be initiated from the age of 40 years or 10 years younger than the age of the first patient diagnosed in a family [22].

According to the Iranian National Cancer Registry Report, the incidence of CRC has increased during the last 25 years showing that the distribution of CRC has shifted towards lower age groups in the country. In addition, Iran still faces challenges regarding the access to anticancer medicines, especially costly anti-*EGFR*-targeted cancer therapy protocols. So despite high cost of molecular techniques, it is obvious that screening of CRCs who are candidates for these therapies by detecting of mutations in* KRAS* and* BRAF* genes is recommended, particularly to those who are not likely to benefit for avoiding unnecessary costs. In general, it is now clear that routine molecular approaches of CRC including* KRAS* and* BRAF* testing are cost saving [23].

To set up an efficient screening procedure, the prevalence of disease, demographic characteristics of the population, possibility of early diagnosis, availability of new treatment modalities for patients with positive screening tests, and the cost-benefit of the whole procedure will have to be determined if risk assessment tool needs to be conducted.

This review study was conducted to document the epidemiologic features of CRC in Iran: age standardized incidence, survival rates, prognostic factors, burden of disease, heredity pattern, occurrence of sporadic cases in relatives, and so forth. We also give a report on the molecular routes and cellular metabolism, mutation in* KRAS* and* BRAF* genes, and other tumor markers in the country.

## 2. Materials and Methods

To retrieve published studies and to identify the studies of interest, we conducted a comprehensive literature search. The databases Medline, Embase, SID (Scientific Information Database), Magiran, Iran Medex, and Google Scholar were searched for English and Persian articles from year 2000 to recently published studies. As for search criterions, we used medical subject headings including colorectal cancer, molecular genetics,* KRAS* mutation,* BRAF* mutation, screening, survival, epidemiologic study, and Iran to retrieve the relevant evidence.

We did a careful review on the full text of the articles retrieved for this study. The reference lists of articles were also reviewed to identify any additional relevant resources on the same search area.

## 3. Results

### 3.1. Descriptive Epidemiology of CRC in Iran

Recent figures published by the Iran Ministry of Health showed that cancer is the third common cause of death in the country. It is now ranked in the third place after heart diseases and accidents with an increasing incidence over the past few decades in the population [24, 25].

The latest data by the Iran National Cancer Registry (INCR) reported an annual number of 51,000 cases of cancer with about 35,000 cancer-related deaths in the country. This is the second highest in the Eastern Mediterranean region according to the World Health Organization reports. The same report shows that CRC was the fourth most common cancer in Iran between 2000 and 2009 [26, 27], with a five-year survival rate between 43 and 49 percent [28–30].

The annual report of the INCR shows that CRC is the fourth common cancer in men after stomach, bladder, and prostate. It is however the second one among women after breast cancer [13, 26, 31, 32]. A research study in 2008 reported an ASR of 7-8 (per 100,000) for both sexes. This is higher than that of previous reports in the country while closer to the statistics reported from other Middle East areas and less than the similar rates in western countries [1, 32]. Safaee et al. in 2012 reported that the overall ASR during a four-year period (2008–2012) was 38 (per 100,000) with higher rates in men (39.96) compared to women (36.16). The overall rate was relatively high compared to other Asian countries. ASR among individuals younger than 50 years was also estimated 8.26 (per 100,000) in the same report. Adenocarcinoma was the most frequently detected type of colorectal carcinoma and most common tumor diagnosed in the colon [33].

An ASR of 5.9 and 10.7 has been reported from female population of Ardebil and males in Golestan, respectively. In each province, CRC is ranked second to fourth representing 6–8 percent of all types of cancers [1]. According to Somi et al. from northwest of the country in 2014, annual ASR was estimated 11.2 and 8.93 in men and women, respectively. The crude rates for men and women were reported 11.5 and 9.22, respectively, in the same study [34]. The occurrence of CRC has recently shown a slightly decreasing trend compared to the 2008 data [31, 35, 36].

CRC is normally a disease of aged people occurring in individuals over 65 years. However, as indicated before, several epidemiological studies in Iran have shown that the proportion of CRC among the younger population is considerably higher compared to western populations. Early CRC (in less than 40 years) accounts for almost one-fifth of all cases of CRC in Iran, compared with high risk countries, with rates ranging from 2 to 8% [1, 13, 37–44]. This might be attributed to two factors: a high proportion of young population in Iran and a relatively low rate of CRC in aged people in the country [13].

Almost all economic indices show a considerable improvement in living conditions in Iran over the past few decades (Statistic Center of Iran, 2007, World Bank report, 2006). This has been accompanied by considerable changes in lifestyle including sedentary lifestyle and a diet rich in fat and meat and poor in grains and fiber [13]. The recent trends of CRC in youth in Iran could be a result of these changes in the diet pattern in this generation towards western lifestyle in recent decades [1]. It is therefore expected that the occurrence of CRC in Iran will probably be similar to those of western countries in the forthcoming decades. Some studies showed that genetic factors may probably play a role in the development of CRC among young population in Iran, as well. This suggests that CRC in Iran could be caused by different factors both environmentally and genetically [33, 45].

### 3.2. Survival Rates of CRC Patients

The prognosis of CRC depends on various factors associated with tumor characteristics, patient condition, diagnosis, and treatment of the disease [19]. Advanced stages of cancer, rectal localization of tumor, pathology grade, and patient's performance, vascular invasion, older age, and genetic instability such as amplification of certain genes are the factors that correlate with the worst prognosis of CRC patients at diagnosis. The surgical technique and proper use of adjuvant and neoadjuvant treatments considerably affect the overall survival of patients [30, 46, 47].

The results of study mentioned by Haghighi et al. showed that overall 5-year survival of HNPCC patients was higher than sporadic ones (*P* = 0.044), and they compared their data with other similar studies. Even patients with sporadic CRC had a risk of death about threefold of those with HNPCC. These differences are probably due to different clinical pathological characteristics of neoplasia and genetic alteration [48, 49].

Survival also varies greatly with the geographical and economic status, host response, the accuracy and availability of diagnostic procedures, and availability of treatment [30]. Poor survival with advancing age was observed in a few studies from Iran, which may be attributed to poor general health condition and limitations of cancer therapies in the elderly [49, 50]. However, the results of a population-based cohort study from Golestan in Iran, where more than 42% of CRCs were under 50 years of age, revealed a worse survival in young CRC patients, more likely because their cancer is diagnosed in advanced stages [50]. Finally, race and ethnicity as measures of socioeconomic status have a major impact on CRC incidence rates, tumor characteristics, mortality rates, and survival rates [51].

A research study showed that the one-, two-, three-, four-, and five-year survival rates of CRC were 84, 68, 54, 43, and 41 percent, respectively. The median overall survival was estimated to be 3.5 years. Men had poor prognosis compared to women where the five-year survival rate was 45 and 88 percent in men and women, respectively. The poorest prognosis was observed in those younger than 20 years and older than 80 years. The largest number of patients belonged to the 50–70 years age group. The overall five-year survival rate for CRC patients in Iran (41 percent) was similar to those from other developing countries while it was lower compared to developed world where advanced health care systems and population-based screening programs exist [30].

### 3.3. Molecular Features of CRC in Iran

The epidermal growth factor receptor (*EGFR*) plays a key role as a receptor of tyrosine kinase (*TK*).* EGFR* mutations have been detected in several types of cancer [50]. A study in Iran on locally advanced rectal cancer has shown the expression of* EGFR* functions as a predictor of response to treatment in these tumors. They can thus be used in the recognition of a subgroup of patients who are likely to be unresponsive to this method of treatment [13].

Mutations in* KRAS* and* BRAF* genes are considered important primary incidents in the development of CRC. It shows strong association with the incidence of methylguanine in promoter regions of various genes in CRC [51]. While* KRAS* has been investigated as a unique marker, simple or multivariate studies did not indicate any effects on progression-free survival (PFS). However, existing evidence indicates that patients with metastatic tumors as well as* KRAS* mutation show worse prognosis than patients with wild-type* KRAS*. Similarly, existing evidence failed to implicate* BRAF* mutation as the sole predetermining factor for PFS. There is, however, sufficient evidence showing that mutation in* BRAF* gene is a negative prognostic marker for overall survival of patients [52].

### 3.4. Mutation in* KRAS* Gene

Mutation in* KRAS* gene is one of the most common oncogenic changes in various types of human cancer [28]. Mutation in* KRAS* gene occurs early in the development of cancer and may therefore play an important role in several stages of cancer progression and development of malignancy including the initiation of neoplasia and metastasis and prediction of prognosis [50, 51]. Mutation in codons 12, 13, and 61 of* KRAS* is common in CRC. This produces an active* KRAS* protein resulting in the activation of the* MAPK* (mitogen activated protein kinase) cascade independently of* EGFR* activation. Recent reports have shown that mutation in codon 12 is observed at frequencies of 12–30 percent and 35–50 percent among CRC patients in Asian and western countries, respectively [51]. Various frequencies of mutations in different populations may be attributable to the effect of environmental factors and food habits as well as genetic factors.* KRAS* mutation is mostly observed in right-side tumors, low-grade or* MSI*-low (microsatellite instability-low) and* MSS* (microsatellite stable) tumors. It does not seem to have an impact on the prognosis or progression-free and overall survival rates [16].

Although* KRAS* mutations have been widely studied in CRCs in western countries, there are few data on* KRAS* mutation and spectrum in CRCs from Iran. A study in Iran by Bishehsari et al. reported 37.4 percent of CRC patients with* KRAS* mutations. This rate is within the range of 25–50 percent reported in studies of other CRC series. Codon 12 was the most frequently mutated (66 percent), followed by codon 13 (32.5 percent), while codon 61 was only mutated in one of 182 tumors in this investigation. This confirms that codons 12 and 13 are preferentially involved in CRC progression in Iranian population, as well. The investigators compared their results of* KRAS* mutations from Iran to those from Italy. They found an overall rate of* KRAS* mutations in the Italian series (46.3 percent) which is higher than that found in Iranian CRCs. Although mutations in codon 12 were similar in both series, codon 13 was more frequently detected in CRCs from Italy [53]. Another study from Iran in 2010 reported that 20.3 percent (12 cases) of CRCs (10 in codon 12 and 2 in codon 13) have shown a point mutation. This is still lower than the similar proportions from other western countries [54].

### 3.5. Mutation in* BRAF* Gene

*BRAF* is a cytoplasmic serine-threonine kinase that mediates cellular response to growth signals of* EGF* through the* RAS/RAF/MAPK* pathway. The activation of* BRAF* gene through mutation has been identified in 5–15 percent of sporadic cases of CRC. More than 80 percent of the mutations are in the hot spot of exon 15 resulting in the* V600E* mutation in codon 600 [55]. Efforts to identify other mutations in tumors that would facilitate the identification of patients who would be more responsive to anti-*EGFR* immunotherapy have revealed that an activating mutation in* BRAF* gene among patients with wild-type* KRAS* is likely to result in a weak response. CRC on the right side along with* BRAF* mutation and microsatellite stability leads to a definite weak prognosis for overall as well as progression-free survival.* BRAF* mutation is often observed in tumors on the right side in patients of 60 years and more, in women, and also in MSI-high or highly malignant tumors [16]. A study in Iran by Naghibalhossaini et al. showed the absence of any BRAF mutation among the Iranian population supporting the observations of Brim et al. that minimum mutation rates of this gene are found among patients in Iran (2 percent) compared to those in Amman (19 percent) and African-Americans (10 percent). A low rate of* BRAF* mutation in CRC was also observed among Asians. Genetic or environmental factors are believed to be responsible for the lack of* BRAF* mutation in CRC patients in Iran [5, 51, 56].

### 3.6. Mutation in* TP53 *Gene

*P53* is a tumor suppressor gene which is believed to play a role in the regulation of cell proliferation, involved in DNA repair, cell cycle gene transcription, and apoptosis. When the DNA damage cannot be successfully repaired,* P53* initiates the apoptosis, and cell dies with the help of cell death genes. Mutations in* TP53* are major genetic alterations involved in CRC progression. Actually, mutation in* P53* is the most frequently detected genetic alteration in most human cancers [8].

*P53 *loss of function is frequently present in the later stages of colorectal cancer.* P53 *also interacts with cytooxygenase-2 (Cox-2), indicating that Cox-2 could be an independent prognostic factor in CRC. The most frequently mutated region of* P53 *gene is exons 5 to 8 of the human* TP53*. Also it is believed that some mutations in this gene lead to resistance to common treatment protocol chemotherapy drugs in CRC patients, so an effective treatment can be planned after* P53* mutation testing [57].

Scattered studies are available on mutation frequency of* P53* among CRCs across Iran. Previous studies showed overexpression of mutant* P53* in 59–63% of patients with colorectal cancer using IHC method (immunohistochemical), while sequencing of amplified DNA samples revealed the rate as 23–44% [58–60]. According to results of Malekzadeh et al.,* P53* gene mutation in Iranian CRCs occurs as frequent as in other series, but proximal and distal side of colon show different* P53* mutation patterns, which may suggest different tumorogenesis pathways of proximal and distal colon [13, 58]. However, it is believed that* P53* alterations are more frequent in distal than in proximal CRCs, and a recent meta-analysis of case series from 17 countries showed that sites and types of* P53* mutations were comparable in proximal and distal colorectal cancers [58, 61].

### 3.7. Mutation in* APC *Gene

Adenomatous polyposis coli (*APC*) gene is a tumor suppressor gene and encodes a cytoplasmic protein which binds to *β*-catenin and it is widely accepted that the* APC* tumor suppressor gene inactivation is the earliest and key event for mutation initiation in more than 80% of early colorectal cancers, and consequently it is named as “Gate Keeper” for adenoma development [2, 7, 14]. The majority of* APC* mutations in the* MCR* (mutation cluster region) introduce a stop codon, resulting in a truncated protein which lacks the binding site for two important interactants, *β*-catenin and axin, which act together in the* Wnt-* (wingless type-) signaling pathway.

The location of mutation seems to be related to disease severity and presence of extracolonic manifestations in FAP patients. There are a few reports about* APC *gene mutation in Iranian FAP patients, which revealed that the mutation pattern is the same as other reports from other countries [62]. In a case series report, Kashfi et al. reported frame shift mutations in exon 15 and two siblings with germline mutation at codon 849 and two FAPs had frame shift mutation at codon 1309 [63]. The study of Kashfi et al. on ten unrelated Iranian FAP patients identified 5 mutations at exon 15 of* APC *gene [63]. It has been reported that about 30% of FAP patients do not have any identifiable germline* APC* mutations [8].

Depending on ethnicity, geographical region, dietary, and genetic predisposition and mostly because of heterogeneous nature of colorectal cancer,* APC *mutation genes are considerably diverse and distinct, as well as in sporadic CRCs. It occurs in 34–80% of sporadic colorectal cancers, and mutation in exon 15 covers more than 75% of coding sequence [64]. To date, some hundred mutations have been reported around the world, but some studies in Iran reported a unique profile of* APC *mutations in Iranian CRCs, and the overall frequency of* APC *mutation was about 25–30% in sporadic colorectal patients [65].

### 3.8. Mismatch Repair (MMR) Genes

The inactivation of* MMR* genes is mostly caused by epigenetic silencing via promoter hypermethylation. The failure of* MMR* genes subsequently leads to mutations in specific target genes involved in proliferation and cellular differentiation.* MMR* genes lead to failure of DNA mismatches repair during replication and generation bases and also encode proteins for correcting DNA nucleotide base mispairs. Microsatellites (*MSI*) are simple DNA sequences which consist of repeating unit of 1–5 bp, with 25–60 bp in length, which are distributed throughout the genome. MSI is detected on an average of 10–15% of sporadic CRCs and is the important underlying event in 85–90% of HNPCCs. The five suitable markers include* BAT25*,* BAT26*,* D2S123*,* D17S250*, and* D5S346* [2].

Several studies showed high frequency of* MSI* in sporadic colorectal cancer patients in Iran, and* BAT25*,* BAT26*, and* NR-21* were showed to be the most sensitive markers for diagnosis sporadic CRCs and HNPCCs [13, 66–69].* MSI* status in Iran was observed in 23% of CRC cases, and it was more frequent in females, in early-onset colorectal cancers, and in tumors located in the proximal colon (40%), with the majority of* MSI-H* tumors, compared to the common trends in the world [13]. Moghbeli et al. detected MSI in about 43% of their patients with 27% of* MSI-H*, while Bishehsari et al. analyzed 170 sporadic CRCs, with 19.4%* MSI-H* [53, 67]. These different frequencies suggest that the molecular epidemiology of genetic alterations involved in the CRC carcinogenesis has varied in the Iranian population [67]. Compared with sporadic CRCs, about 59% of HNPCCs have shown* MSI* in* BAT26* and* BAT25* from one report by Galehdari et al., which was considered lower than other previous findings. There was no significant relation to the stage and location in comparison with both markers, and all of them were* MSI*-low phenotype [68]. In another survey on 32 HNPCC Iranian families, mutation of* MLH1* and* MSH2* genes was identified in about 63% of their patients [70].

Despite increasing knowledge about genetics and biochemistry of the* MMR* genes, a few data about mutation screening of* MMR* in Iranian CRCs, as well as HNPCC patients, are available. Indeed, while the detection of* MSI* is simple and economical and has high correlation with the clinicopathologic feature of colorectal carcinoma, large and more executive molecular research studies are needed to know about factors that contributed to* MSI*-associated carcinogenesis in Iranian CRCs.

### 3.9. Tumor Markers in Iranian CRC

Carcinoembryonic antigen (CEA), a serum biomarker, is routinely used in clinical practice and is considered a major tool for the control of cancer metastasis to the liver. Since its identification in colon adenocarcinoma in 1965, CEA has been shown to play an important role in the attachment of cancer cells [71]. The marker shows association with disease stage, and the levels of CEA and CA19-9 before surgery are correlated with the disease prognosis. After the removal of the primary tumor, serum levels of CEA and CA19-9 are serially measured every 2-3 months for at least 2 years for obtaining information about the cancer or its metastasis. However, no consensus has been reached regarding the extent of changes in CEA levels in the assessment of higher probability of cancer onset or progression [16]. Also other findings showed that the CEA level may be a suitable biomarker for predicting tumor response to specific chemotherapy in colorectal cancer [72].

### 3.10. Heredity and CRC Screening

The high occurrence of CRC in records of relatives of CRC patients in Iran shows that a considerable number of these CRC patients are from families whose members or relatives have been suffering from CRC in the past. Malekzadeh et al. showed that family history of CRC is more common among young CRC patients and with tumors on the right side [13]. Moreover, the incidence of CRC in a close family member during their youth leads to considerably increased risk of acquiring CRC (15–30 percent) [18]. Familial risk of colorectal cancer has been described in a number of publications. A cohort study of 32,085 men and 87,031 women conducted by Fuchs et al. (1994) provided baseline data on first degree relative with CRC, diet, and other risk factors. They revealed that the familial relative risk in first degree relatives of CRCs was about 1.7, rising to 2.8 if two or more first degree relatives were affected or/and to 5.4 if both relatives were less than 45 years old at diagnosis [73].

Studying the colonoscopy results, in a few studies in Iran, indicated that having a family member with a history of CRC, 3–2.7-fold, increases the risk of colorectal polyps and CRC [74–76]. Also the incidence of adenoma among the relatives of CRC patients aged 50–60 years is approximately 17–34 percent and lower among younger relatives [32, 77]. Another study's results showed that the prevalence of colorectal adenoma and precancerous lesions in first degree relatives of CRC patients is significantly higher than in the average risk population [77]. According to different studies, one in every 6 (16 percent) and one in every 5 (20 percent) CRC patients have a first degree relative (either a parent or sibling) suffering from CRC. Studies have shown that the incidence of CRC in a close member of one's family almost doubles the risk of CRC for an individual and the risk of developing CRC is 2.5–3 times greater among the relatives of CRC patients [19]. These observations highlight the need to improve efforts for enhancing public awareness and screening strategies in families with a CRC patient, particularly a young patient, or with proximal tumors. Screening programs may therefore begin with family members of CRC patients from a young age using colonoscopy as the preferred method of screening [13].

### 3.11. Implications for Screening of CRC

The increased ASR observed beyond 50 years of age suggests that screening from 50 years of age onward might be appropriate for high risk groups. However, all characteristics of CRC and differences unique to the given region should also be taken into account. Several studies have assessed the efficiency of CRC screening in individuals aged 50 years or below [33]. A timely diagnosis and the removal of adenomatous polyps may help the prevention of CRC. The diagnosis of local CRC may also improve the survival rate of CRC patients, and the five-year survival rate of CRC diagnosed early was reported to be around 90 percent [75]. Often, CRC is caused by adenomatous polyps, whose sizes vary from small (less than 5 mm) to large (more than 1 cm) and also vary between dysplasia and cancer. This development route of cancer from dysplasia probably lasts at least 10 years for a large number of individuals [32, 77].

Although the early diagnosis of CRC reduces the associated death burden, administration of screening programs is not a common practice even in developed countries. Previous studies showed the absence of any national screening programs for CRC in Iran, with very little information available on screening strategies for cancer in general in the country [78]. In fact, a few screening programs were tested among the high risk groups for CRC, which include family members of CRC patients, by the Center for Liver and Digestive System Studies in the Medical Science and Medical Services Department of Shahid Beheshti University [77, 79]. However, some educational programs on CRC screening may increase overall knowledge and motivation about colorectal cancer risk factors and screening modalities [79].

On the whole, screening of even average risk groups for CRC, who are exposed to less than acceptable risk levels, is economical. The decision making involved in CRC screening in Iran has proven valuable for cost saving with respect to local resources, personal preferences of the patients, or the choice of screening tests, including the highly sensitive stool occult blood test conducted annually (such as stool immunochemistry test) or colonoscopy conducted every 10 years. Colonoscopy is both the most effective and sensitive and the most costly strategy according to latest trials in Iran [25, 77, 80, 81], and many studies recommend it as golden standard tool for early detection of precancerous as well as CRC [77, 82]. As well as pathology findings, genetic history and information content on pedigree play a significant role in planning and management of colorectal cancer screening [79].

In fact, because of lack of comprehensive studies about screening programs in Iran, we can only recommend screening program and colonoscopy as a golden standard every 5 to 10 years, for people with increased risk and high risk syndromes. Colorectal cancer increases significantly beyond the 5th decade of life; therefore, it is often thought of as a disease of the elderly, and CRC screening is usually not recommended for individuals at average risk younger than 50 years [3]. Increased risk patients include as above the following:

- personal history of adenoma, CRC, and inflammatory bowel disease (ulcerative colitis, Crohn's disease),
- positive family history (particularly first degree relative with CRC).

High risk syndromes include the following:

- Lynch syndrome (hereditary nonpolyposis colorectal cancer (HNPCC)),
- polyposis syndromes (classical familial adenomatous polyposis (FAP), attenuated familial adenomatous polyposis (AFAP),* MUTYH*-associated polyposis (MAP), Peutz-Jeghers syndrome, juvenile polyposis syndrome, and serrated polyposis syndrome)
- Cowden syndrome,
- Li-Fraumeni syndrome.

## 4. Conclusion

Similar to other screening programs, the strategy for screening for CRC should be assessed with respect to its effectiveness, sensitivity, the number of false positive results, safety, and comfort. Furthermore, the cost and economic factors pertaining to the screening programs should be observed in order to help patients with decision making, and the prevailing clinical policies should be taken into consideration.

However, the devising and administration of screening programs in every country requires basic epidemiological information, including assessments of the severity of cancer prevalence, the average- and high risk groups for CRC, the most common tumor localization, age distribution of the patients at the time of diagnosis, and calculation of the attributed risk of CRC and a high performance risk assessment tool. Whereas the efficacy of screening is clearly useful for people with increased and average risk and high risk syndromes, there is no evidence that specific mass screening programs on adolescent and young adults in Iran would increase early detection and impact on survival, so more comprehensive and general studies are required to prove this claim. Besides, it should be determined which method of screening gives better outcomes. The development of an executive plan to identify the most appropriate screening method and the best age group to be screened for CRC is highly recommended in Iran.
